# Complementary feeding practices and associated factors among mothers of children aged 6 to 23 months in Sub-saharan African countries: a multilevel analysis of the recent demographic and health survey

**DOI:** 10.1186/s12889-023-17629-w

**Published:** 2024-01-08

**Authors:** Enyew Getaneh Mekonen, Alebachew Ferede Zegeye, Belayneh Shetie Workneh

**Affiliations:** 1 Department of Surgical Nursing, School of Nursing, College of Medicine and Health Sciences, University of Gondar, Gondar, Ethiopia; 2 Department of Medical Nursing, School of Nursing, College of Medicine and Health Sciences, University of Gondar, Gondar, Ethiopia; 3 Department of Emergency and Critical Care Nursing, School of Nursing, College of Medicine and Health Sciences, University of Gondar, Gondar, Ethiopia

**Keywords:** Complementary feeding practices, Young children, Sub-saharan Africa

## Abstract

**Introduction:**

Malnutrition is a public health problem in sub-Saharan Africa with an increased morbidity and mortality rate than in other parts of the world. Poor complementary feeding practices are one of the major causes of malnutrition during the first two years of life. Therefore, this study aimed to determine the prevalence and associated factors of appropriate complementary feeding practices among mothers of children aged 6 to 23 months in sub-Saharan African countries.

**Methods:**

A multilevel mixed-effect analysis was carried out using recent demographic health survey data from 19 sub-Saharan African countries, which were conducted between 2015 and 2020. A total weighted sample of 60,266 mothers of children aged 6 to 23 months were included in the study. The demographic health survey employs a stratified two-stage sampling technique. Data extracted from the recent DHS data sets were cleaned, recorded, and analyzed using STATA/SE version 14.0 statistical software. Multilevel mixed-effects logistic regression was used to determine the factors associated with complementary feeding practice. Variables with a *p*-value less than 0.05 and adjusted odds ratio (AOR) with a 95% confidence interval (CI) were reported as statistically significant variables associated with appropriate complementary feeding practices.

**Results:**

The prevalence of appropriate complementary feeding practices among mothers of children aged 6 to 23 months in sub-Saharan African countries was 13.02% (95% CI: 12.75–13.29%). Maternal educational level [AOR = 0.69, 95% CI (0.64, 0.74)] and [AOR = 0.52, 95% CI (0.47, 0.57)], marital status of the mother [AOR = 0.85, 95% CI (0.74, 0.96)], sex of household head [AOR = 1.78, 95% CI (1.09, 1.27)], total children ever born [AOR = 1.52, 95% CI (1.18, 1.96)], [AOR = 1.43, 95% CI (1.14, 1.81)], and [AOR = 1.31, 95% CI (1.04, 1.64)], media exposure [AOR = 0.74, 95% CI (0.69, 0.79)], ANC visits attended during pregnancy [AOR = 0.73, 95% CI (0.63, 0.80)] and [AOR = 0.67, 95% CI (0.62, 0.74)], place of delivery [AOR = 0.92, 95% CI (0.85, 0.98)], currently breastfeeding [AOR = 1.12, 95% CI (1.01, 1.23)], PNC checkup [AOR = 0.75, 95% CI (0.70, 0.80)], the current age of the child [AOR = 0.26, 95% CI (0.24, 0.28)] and [AOR = 0.14, 95% CI (0.13, 0.16)], birth order [AOR = 1.31, 95% CI (1.09, 1.58)], number of under 5 children in the household [AOR = 0.76, 95% CI (0.59, 0.97)], community illiteracy [AOR = 1.09, 95% CI (1.02, 1.18)], and country category [AOR = 1.62, 95% CI (1.18, 2.22)] were significantly associated with appropriate complementary feeding practices.

**Conclusion:**

The prevalence of appropriate complementary feeding practices among mothers of children aged 6 to 23 months in sub-Saharan Africa was relatively low. Higher maternal educational level, female household head, having media exposure, attending more ANC visits, health facility delivery, currently breastfeeding, having PNC follow-up, low community illiteracy, and living in the West Africa region increase the odds of appropriate complementary feeding practices. Women empowerment, increasing maternal health services accessibility, promoting breastfeeding behavior, increasing media exposure of the household, and improving the proportion of health facility delivery are strongly recommended.

## Introduction

Malnutrition is a public health problem in sub-Saharan Africa, with an increased morbidity and mortality rate more than in other parts of the world [1]. It affects one-third of the population, with short- and long-term impacts including mental and physical growth retardation, immunosuppression, diseases, and greater risks to health and the future [2]. Undernutrition during the first 32 months of post-conception affects the cognitive and physical health of the child and the economic productivity of nations, which results in lower academic performance, poor economic productivity in adulthood, and social problems [3–5]. Poor dietary intake is the common cause of long-term malnutrition among children [6]. During the first two years of life, the major causes of malnutrition are poor complementary feeding (CF) practices and breastfeeding, in combination with a high burden of infectious diseases [7].

Adequate nutrition is fundamental during the period from birth to 24 months of age, as it is a “critical window” for the advancement of ideal growth, health, and behavioral change [7]. Complementary feeding is the consumption of other foods and liquids when breast milk alone is no longer adequate to meet the nutritional necessities of infants [8]. It must provide optimal macro- and micronutrient intake to endorse appropriate health outcomes in early and later life, in addition to meeting the optimal growth and development of infants [9]. Optimal CF during the first 2 years of life is important since rates of malnutrition usually peak at this time and the damage to physical growth and brain development is irreversible [10]. Adequate CF is the key to addressing the rapidly increasing nutrient gap between 6 and 24 months that contributes to undernutrition and its consequences, including stunting and wasting [11].

Inappropriate CF practices like late or early initiation of complementary food, incorrect feeding frequency, and low dietary diversity are associated with different negative effects on infant and young children’s health [12]. It has been identified as the commonest cause of diarrhea, malnutrition outcomes, and mortality [13]. The prevalence of stunting among under-five children in sub-Saharan Africa (SSA) was 41%, and poor feeding practices were an important risk factor [14]. Inappropriate CF is also responsible for 6% of child mortality in the region [15].

Less than half of children aged 6–23 months meet the feeding frequency and dietary diversity in most countries [16]. The prevalence of appropriate CF practice among mothers of children aged 6–23 months was 51.3% in India [17], 4.2–10.0% in Nigeria [18, 19], 15.7–29.8% in Ghana [20, 21], 7.7% in Malawi [22], and 9.76% in Ethiopia [23]. The child’s age, education status of mothers, wealth index, ANC visit, current breastfeeding mothers, residence, birth order, and mother’s workplace were determinants of appropriate CF practice from previous studies conducted in different areas [17–20, 23].

As far as the researchers’ knowledge is concerned, CF practice in SSA has not yet been investigated using the revised indicators for assessing infant and young child feeding practices [24]. Identification of factors associated with CF practice in multiple countries using the Demographic and Health Survey (DHS) in this sub-region can help develop shared strategies to deal with infant and young child feeding practices in SSA. Therefore, this study was intended to assess the prevalence and associated factors of appropriate CF practices among mothers of children aged 6 to 23 months in SSA countries using recent DHS data. The findings of this study will provide insights into the development of effective strategies by health policymakers, planners, and other organizations regarding the CF practice of children aged 6 to 23 months in this sub-region.

## Methods and materials

### Study design, period, and setting

A multilevel mixed-effects analysis was carried out using recent DHS data from 19 sub-Saharan African countries, which was conducted between 2015 and 2020. The DHS is a community-based cross-sectional study that is conducted every five years to produce updated population and health-related indicators [25].

### Study participants

All sub-Saharan African mothers of children aged 6 to 23 months were the source population of the study. Mothers of children aged 6 to 23 months living in the randomly selected enumeration areas of each country during the survey year were the study population of this study.

### Data sources

For this secondary data analysis, the most recent sub-Saharan African countries’ DHS datasets from 2015 to 2020 were used. We used DHS surveys from 19 sub-Saharan African countries, including Angola, Benin, Burundi, Cameron, Ethiopia, Gambia, Guinea, Liberia, Mali, Malawi, Nigeria, Rwanda, Serra Leone, Senegal, Tanzania, Uganda, South Africa, Zambia, and Zimbabwe. The data were appended to figure out the prevalence and associated factors of appropriate CF practice among mothers of children aged 6 to 23 months in sub-Saharan African countries. The survey for each country contains different datasets, including those for children, males, women, births, and households. The DHS is a nationwide survey mostly collected every five years across low- and middle-income countries. It uses standard procedures for sampling, questionnaires, data collection, cleaning, coding, and analysis, which allows for cross-country comparison [25].

### Sample size and sampling procedure

A total weighted sample of 60,266 mothers of children aged 6 to 23 months was included in the study (Table 1). The DHS employs a stratified, two-stage sampling technique [26]. The first stage involves the development of a sampling frame, consisting of a list of primary sampling units (PSUs) or enumeration areas (EAs), which covers the entire country and is usually developed from the latest available national census. The second stage is the systematic sampling of households listed in each cluster, or EA. Further information on the survey sampling strategies is available in the DHS guideline [27].

**Table 1:** Sample size for the prevalence and associated factors of appropriate CF practices among mothers of children aged 6 to 23 months in Sub-Saharan African countries

Country	Year of survey	Weighted sample (n)	Weighted sample (%)
Angola	2015	4,207	6.98
Benin	2017/18	4,018	6.67
Burundi	2016/17	3,973	6.59
Cameroon	2018	2,677	4.44
Ethiopia	2016	2,919	4.84
Gambia	2019	2,372	3.94
Guinea	2018	1,961	3.25
Liberia	2019/20	1,557	2.58
Mali	2018	2,810	4.66
Malawi	2015	4,843	8.04
Nigeria	2018	9,139	15.16
Rwanda	2019/20	2,370	3.93
Serra Leone	2019	2,697	4.48
Senegal	2019	1,827	3.03
Tanzania	2015	3,136	5.20
Uganda	2016	4,359	7.23
South Africa	2016	891	1.48
Zambia	2018	2,853	4.73
Zimbabwe	2015	1,657	2.75
Total sample size		60, 266	100

### Variables of the study

#### Dependent variable

complementary feeding practices (appropriate or inappropriate).

#### Independent variables

since demographic and health survey data are hierarchical, independent variables from two sources (variables at the individual and community levels) were taken into consideration for this analysis.

#### Individual-level variables

this level includes maternal socio-demographic factors (maternal age, maternal education, marital status, sex of household head, total children ever born, ownership of a mobile telephone, media exposure, wealth index), maternal health service and related factors (parity, ANC visits attended during pregnancy, place of delivery, current breastfeeding status, PNC checkup), and child-related factors (sex of the child, age of the child, birth order, preceding birth interval, and the number of under-five children in the household) of the study.

#### Community-level variables

place of residence (urban or rural), community illiteracy (low or high), community-level poverty (low or high), community media exposure (low or high), and country category (Central, Eastern, West, and Southern sub-Saharan).

### Operational definitions

#### Appropriate complementary feeding practices

infants and young children feeding practices that satisfy the minimum dietary diversity (MDD), minimum meal frequency (MMF), minimum acceptable diet (MAD), and introduction of solid, semi-solid, or soft foods (SSSFs) at the recommended diversity, frequency, and time. It was measured using the composite indicators (MDD, MMF, MAD, and SSSF) recommended by the WHO [24].

Introduction of SSSFs: the proportion of youngest children aged 6–8 months who are living with their mother who consumed solid, semi-solid, or soft foods in the 24 h preceding the interview.

Minimum dietary diversity: the proportion of children aged 6–23 months who consumed foods and beverages five or more out of the eight defined food groups during the day or night preceding the survey. The eight food groups are breast milk; grains, roots, and tubers; legumes and nuts; dairy products (infant formula, milk, yogurt, and cheese); flesh foods (meat, fish, poultry, and liver or organ meats); eggs; vitamin A-rich fruits and vegetables; and other fruits and vegetables.

Minimum meal frequency: The proportion of children aged 6–23 months who consumed solid, semi-solid, or soft foods (but also including milk feeds for non-breastfed children) at least the minimum number of times in the 24 h preceding the interview. The minimum number of times is defined as two or more solid, semi-solid, or soft feeds for breastfeeding children aged 6–8 months; three or more solid, semi-solid, or soft feeds for breastfeeding children aged 9–23 months; or four or more solid, semi-solid, or soft or milk feeds for non-breastfeeding children aged 6–23 months, where at least one of the feeds must be a solid, semi-solid, or soft feed.

Minimum acceptable diet: the proportion of children aged 6–23 months who consumed a MAD during the day or night preceding the survey. The MAD is defined as for breastfed children: receiving at least the MDD and MMF as above; or for non-breastfed children: receiving at least the MDD but excluding the dairy products category and MMF and two or more milk feeds.

#### Inappropriate complementary feeding practices

infants and young children feeding practices that did not satisfy one of the above criteria of WHO.

### Statistical analysis

Data extracted from the recent DHS data sets were cleaned, recorded, and analyzed using STATA/SE version 14.0 statistical software. To manage sampling errors and non-responses, sample weight was applied. Descriptive statistics were employed to summarize both the individual and community-level variables. The DHS data’s variables were organized in clusters; children are nested within households, and households are nested within clusters. The assumptions of independent observations and equal variance across clusters were broken to employ the traditional logistic regression model. This is an indication that using a sophisticated model to take into account between-cluster factors is necessary. As a result, factors associated with complementary feeding practice were determined using multilevel mixed-effects logistic regression. Multilevel mixed-effects logistic regression follows four models: the null model (outcome variable only), mode I (only individual-level variables), model II (only community-level variables), and model III (both individual and community-level variables). The model without independent variables (the null model) was used to check the variability of CF practice across the cluster. The association of individual-level variables with the outcome variable (Model I) and the association of community-level variables with the outcome variable (Model II) were assessed. In the final model (Model III), the association of both individual and community-level variables was fitted simultaneously with the outcome variable (complementary feeding practices).

The magnitude of the clustering effect and the degree to which community-level factors explain the unexplained variance of the null model were quantified by checking the intra-class correlation coefficient (ICC) and proportional change in deviance (PCV). A model with the lowest deviance was selected as the best-fitted model. Finally, variables with a *p*-value less than 0.05 and an adjusted odds ratio (AOR) with a 95% confidence interval (CI) were reported as statistically significant variables associated with appropriate CF practices. The existence of multi-collinearity between covariates was checked by using a variance inflation factor (VIF) falling within acceptable limits of 1–10, indicating the absence of significant collinearity across explanatory variables.

### Random effects

The median odds ratio (MOR), ICC, and PCV were used to estimate random effects or measures of variation of the outcome variable. The variation between clusters was measured by the ICC and PCV. Taking clusters as a random variable, the ICC reveals the variation of complementary feeding practices between clusters which is computed as; $ICC=\frac{VC}{VC+3.29}\times 100\%$. The MOR is the median value of the odds ratio between the area of the highest risk and the area of the lowest risk for complementary feeding practices when two clusters are randomly selected, using clusters as a random variable; MOR = 𝑒*e*^0.95√VC^. In addition, the PCV demonstrates the variation in the prevalence of appropriate complementary feeding practices explained by factors and computed as$; PCV=\frac{Vnull-VC}{Vnull}\times 100\%$; where Vnull = variance of the null model and VC = cluster level variance [28–30]. The association between the likelihood of complementary feeding practices and individual and community-level independent variables was estimated by the fixed effects.

## Results

### Socio-demographic, information-related, and economic characteristics of mothers

A total of 60,266 mothers of children aged 6–23 months were included in this study. The mean age of mothers was 28.03 ± 0.03, and 46% of them fall in the age range of 25–34 years. Nearly 37% of mothers had no formal education, and 71% of them were married. The household head was male for 80% of mothers, and only 2% of them had more than nine children ever born. More than half (56%) of the mothers owned a mobile telephone, and 63% of them had media exposure. More than two-thirds (69.4%) of the mothers were from rural areas of sub-Saharan African countries, and 47% of them were poor in terms of their wealth index. More than half (51.74%) of mothers living in SSA countries had poor community media exposure, and 53.08% of them had high community poverty. About 26,381 (43.77%) of the mothers were from western SSA countries and, 47% of them had high community illiteracy (Table 2).

**Table 2:** Socio-demographic, information-related, and economic characteristics of mothers of children aged 6–23 months in sub-Saharan African countries (*n* = 60, 266)

Variables	Category	Frequency (n)	Percentage (%)
**Individual level variables**			
Maternal age	15–24 years	20,703	34.35
	25–34 years	27,696	45.96
	35–49 years	11,867	19.69
Maternal educational level	No formal education	22,005	36.51
	Primary	20,755	34.44
	Secondary and higher	17,506	29.05
Marital status of the mother	Never married	4,545	7.54
	Currently married	42,743	70.92
	Formerly/ever married	12,978	21.53
Sex of household head	Male	48,027	79.69
	Female	12,239	20.31
Total children ever born	1–3	34,349	57.00
	4–6	18,360	30.46
	7–9	6,335	10.51
	> 9	1,222	2.03
Owned a mobile telephone	Yes	26,439	43.87
	No	33,827	56.13
Media exposure	Yes	37,849	62.80
	No	22,417	37.20
Wealth index	Poor	28,278	46.92
	Middle	12,211	20.26
	Rich	19,777	32.82
Community level variables			
Place of residence	Rural	41,827	69.40
	Urban	18,439	30.60
Community media exposure	Low	31,179	51.74
	High	29,087	48.26
Community poverty	Low	28,278	46.92
	High	31,988	53.08
Community illiteracy	Low	31,979	53.06
	High	28,287	46.94
Country category	Eastern	26,110	43.33
	Central	6,884	11.42
	Southern	891	1.48
	Western	26,381	43.77

### Maternal health services and related factors in Sub-Saharan African countries

More than half (53%) of the mothers were multiparous, and nearly 59% of them attended four or more ANC visits during pregnancy. Home delivery among mothers of children aged 6–23 months in sub-Saharan African countries was 28.3%. More than three-fourths (79%) of the mothers currently breastfeed their child, and more than two-thirds (67%) of them had PNC checkups (Table 3).

**Table 3:** Health service and related factors of mothers of children aged 6–23 months in sub-Saharan African countries (*n* = 60, 266)

Variables	Category	Frequency (n)	Percentage (%)
Parity	Primiparous	12,859	21.34
	Multiparous	31,875	52.89
	Grand multiparous	15,532	25.77
ANC visits attended during pregnancy	None	6,819	11.32
	1–3 visits	18,118	30.06
	4 or more visits	35,329	58.62
Place of delivery	Home	17,036	28.27
	Health facility	43,230	71.73
Current breastfeeding status	No	12,691	21.06
	Yes	47,575	78.94
PNC checkup	No	38,821	66.82
	Yes	19,278	33.18

ANC = Antenatal Care; PNC = Postnatal Care

### Child-related factors

More than half (50.73%) of the children were male, and 35.29% of them were aged 12–17 months. The birth order of the child was second to fourth for 48.32% of them, and the preceding birth interval was ≥ 24 months for the majority (87.25%) of the child. About 44,146 (73.25%) of the mothers had 1–2 under five children in the household (Table 4).

**Table 4:** Characteristics of children aged 6–23 months in sub-Saharan African countries (*n* = 60, 266)

Variables	Category	Frequency (n)	Percentage (%)
Sex of the child	Male	30,573	50.73
	Female	29,693	49.27
The current age of the child (in months)	6–11	20,665	34.29
	12–17	21,269	35.29
	18–23	18,332	30.42
Birth order	First-born	13,328	22.12
	Second to fourth	29,123	48.32
	Fifth or more	17,815	29.56
Preceding birth interval (in months)	< 24	7,683	12.75
	≥ 24	52,583	87.25
Number of under 5 children in the household	No child	698	1.16
	1–2	44,146	73.25
	3 and above	15,422	25.59

### Prevalence of appropriate complementary feeding practices

**Fig. 1:**
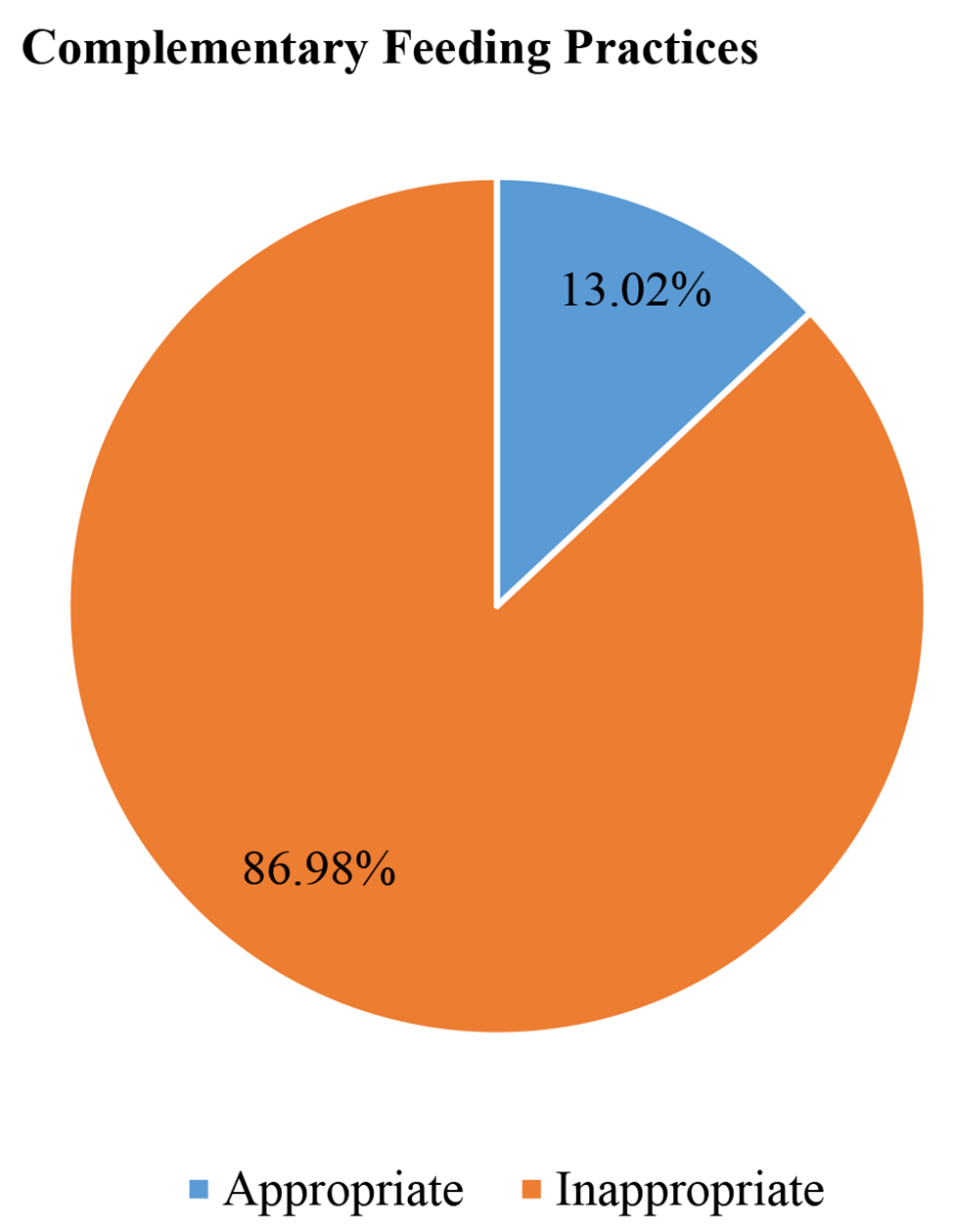
Prevalence of appropriate complementary feeding practices among mothers of children aged 6–23 months in sub-Saharan African countries (*n* = 60, 266)

**Fig. 2:**
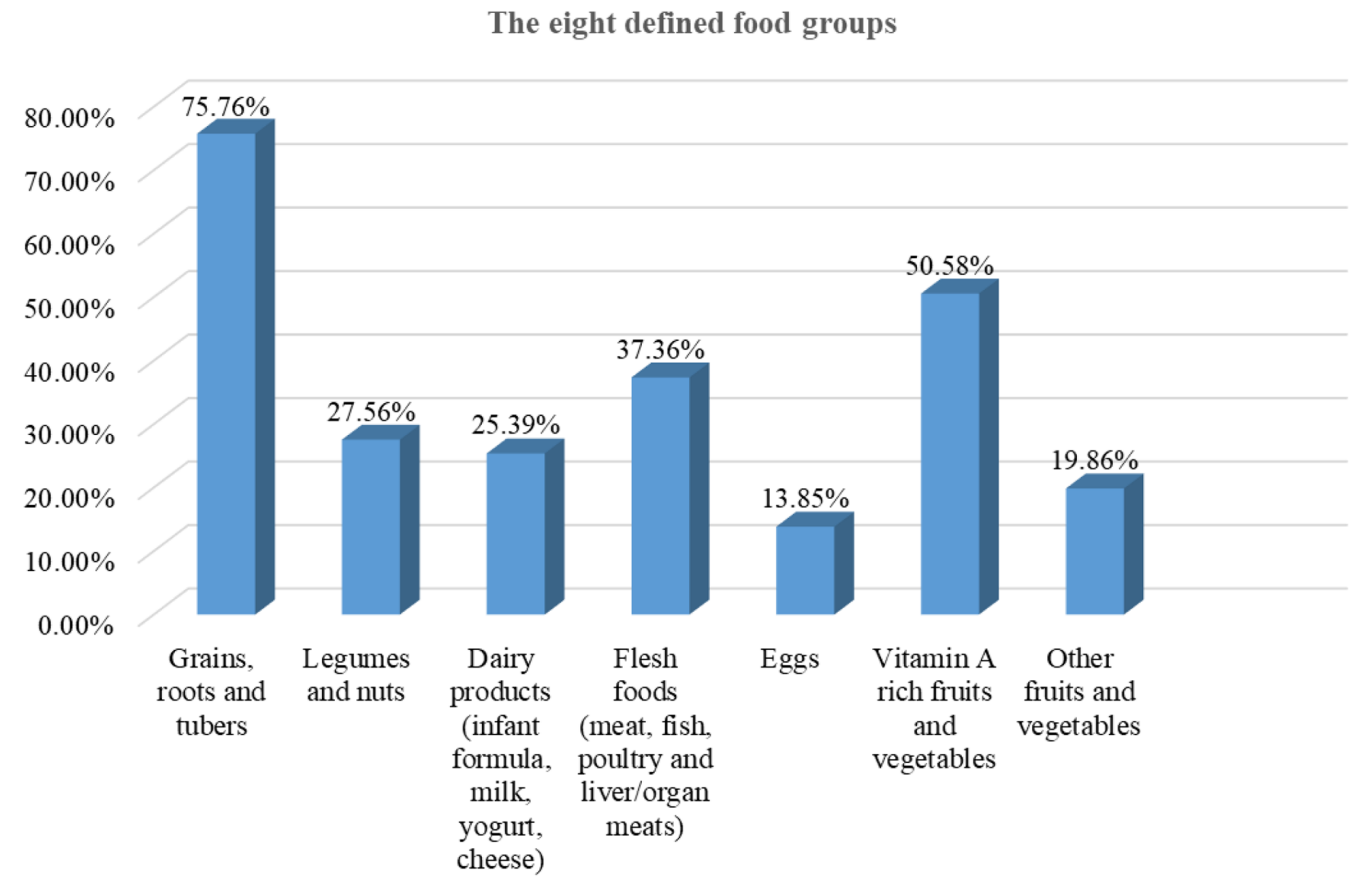
Children aged 6–23 months who consumed from the eight defined food groups during the day or night preceding the survey in sub-Saharan African countries (*n* = 60, 266)

The prevalence of appropriate complementary feeding practices among mothers of children aged 6–23 months in sub-Saharan African countries was 13.02% ((95% CI: 12.75–13.29%) (Fig. 1). The majority (85%) of infants aged 6–8 months enrolled in the study consumed solid, semi-solid, or soft foods (SSSF) in the 24 h preceding the interview. Out of the total children aged 6–23 months included in the study, only 14% and 20% of them consumed eggs and other fruits and vegetables during the day or night preceding the survey, respectively (Fig. 2).

### Random effects (measures of variation) and model fitness

Whether the data supported the decision to assess randomness at the community level was determined by a null model. Findings from the null model showed that there were significant differences in complementary feeding practices between communities, with a variance of 0.0325279 and a *P* value of 0.000. The variance within clusters contributed 90.2% of the variation in complementary feeding practices, while the variance across clusters was responsible for 9.8% of the variation. In the null model, the odds of complementary feeding practices differed between higher and lower risk clusters by a factor of 1.19 times. The intra-class correlation value for Model I indicated that 22.03% of the variation in complementary feeding practices accounts for the disparities between communities. Then, with the null model, we used community-level variables to generate Model II. According to the ICC value from Model II, cluster variations were the basis for 19.82% of the differences in complementary feeding practices. In the final model (model III), which attributed approximately 9.82% of the variation in the likelihood of complementary feeding practices to both individual and community-level variables, the likelihood of complementary feeding practices varied by 0.66 times across low and high complementary feeding practices clusters (Table 5).

**Table 5:** Model comparison and random effect analysis for complementary feeding practices among mothers of children aged 6–23 months in Sub-Saharan African countries

Parameter	Null model	Model I	Model II	Model III
Variance	0.0325279	0.0741018	0.0260805	0.0644603
ICC	9.80%	22.03%	7.86%	19.20%
MOR	1.19	1.29	0.42	0.66
PCV	Reference	12.78%	19.82%	9.82%
**Model fitness**				
LLR	-23295.845	-17027.845	-22999.214	-16776.873
Deviance	46,591.69	34,055.69	45,998.428	33,553.746

ICC: Intra cluster correlation, LLR: log-likelihood ratio, MOR: median odds ratio, PCV: proportional change in variance

### Individual and community-level factors associated with appropriate complementary feeding practices

In the final fitted model of multivariable multilevel logistic regression, maternal educational level, marital status of the mother, sex of household head, total children ever born, media exposure, ANC visits attended during pregnancy, place of delivery, currently breastfeeding, PNC checkup, the current age of the child, birth order, number of under five children in the household, community illiteracy, and country category were significantly associated with appropriate complementary feeding practices among mothers of children aged 6–23 months.

Mothers who had no formal education were 31% and 48% times less likely to have appropriate complementary feeding practices compared with mothers who completed primary and secondary or higher education, respectively [AOR = 0.69, 95% CI (0.64, 0.74)] and [AOR = 0.52, 95% CI (0.47, 0.57)]. Mothers who never married were 15% less likely to have appropriate complementary feeding practices than mothers who were formerly or ever married [AOR = 0.85, 95% CI (0.74, 0.96)]. The odds of appropriate complementary feeding practices were 1.78 times higher among female household heads compared with their counterparts [AOR = 1.78, 95% CI (1.09, 1.27)]. Mothers who had 1–3, 4–6, and 7–9 children ever born were 1.52, 1.43, and 1.31 times more likely to have appropriate complementary feeding practices compared with mothers who had > 9 children ever born, respectively [AOR = 1.52, 95% CI (1.18, 1.96)], [AOR = 1.43, 95% CI (1.14, 1.81)], and [AOR = 1.31, 95% CI (1.04, 1.64)]. Mothers who had no media exposure were 26% less likely to have appropriate complementary feeding practices compared with mothers who had [AOR = 0.74, 95% CI (0.69, 0.79)].

Mothers who did not attend ANC visits during pregnancy were 27% and 33% times less likely to have appropriate complementary feeding practices compared with mothers who attended 1–3 and 4 or more ANC visits, respectively [AOR = 0.73, 95% CI (0.63, 0.80)] and [AOR = 0.67, 95% CI (0.62, 0.74)]. Mothers who delivered at home were 8% less likely to have appropriate complementary feeding practices compared with mothers who delivered at a health facility [AOR = 0.92, 95% CI (0.85, 0.98)]. The odds of appropriate complementary feeding practices were 1.12 times higher among mothers who currently breastfeed their child compared with mothers who didn’t [AOR = 1.12, 95% CI (1.01, 1.23)]. Mothers who had no PNC checkup were 25% less likely to have appropriate complementary feeding practices compared with mothers who had [AOR = 0.75, 95% CI (0.70, 0.80)].

Children aged 6–11 months were 74% and 86% times less likely to have appropriate complementary feeding practices compared with children aged 12–17 and 18–23 months, respectively [AOR = 0.26, 95% CI (0.24, 0.28)] and [AOR = 0.14, 95% CI (0.13, 0.16)]. The odds of appropriate complementary feeding practices were 1.31 times higher among children with a birth order of second to fourth compared with first-borne children [AOR = 1.31, 95% CI (1.09, 1.58)]. Mothers who had no under five children in the household were 24% less likely to have appropriate complementary feeding practices compared with mothers who had 1–2 under five children [AOR = 0.76, 95% CI (0.59, 0.97)]. Low community illiteracy increases the odds of having appropriate complementary feeding practices by 1.09 times [AOR = 1.09, 95% CI (1.02, 1.18)]. The odds of appropriate complementary feeding practices were 1.62 times higher among mothers living in western Africa compared with mothers living in southern Africa [AOR = 1.62, 95% CI (1.18, 2.22)] (Table 6).

**Table 6:** Multivariable multilevel logistic regression analysis of individual-level and community-level factors associated with appropriate complementary feeding practices among mothers of children aged 6–23 months in SSA countries, DHS 2015–2020

Variables	Category	Model I AOR (95% CI)	Model II AOR (95% CI)	Model III AOR (95% CI)
Maternal age	15–24 years	1		1
	25–34 years	0.93 (0.86, 1.01)		0.96 (0.89, 1.04)
	35–49 years	0.92 (0.82, 1.03)		0.94 (0.84, 1.06)
Maternal educational level	No formal education	1		1
	Primary	0.52 (0.49, 0.56)*		0.69 (0.64, 0.74)*
	Secondary and higher	0.45 (0.41, 0.49)*		0.52 (0.47, 0.57)*
Marital status of the mother	Never married	1		1
	Currently married	0.91 (0.81, 1.03)		0.88 (0. 78, 1.00)
	Formerly/ever married	0.79 (0.70, 0.90)*		0.85 (0.74, 0.96)*
Sex of household head	Male	1		1
	Female	1.11 (1.03, 1.20)*		1.78 (1.09, 1.27)*
Total children ever born	1–3	1.58 (1.22, 2.03)*		1.52 (1.18, 1.96)*
	4–6	1.50 (1.18, 1.89)*		1.43 (1.14, 1.81)*
	7–9	1.35 (1.08, 1.70)*		1.31 (1.04, 1.64)*
	> 9	1		1
Owned a mobile telephone	Yes	1.12 (1.05, 1.20)*		1.01 (0.94, 1.08)
	No	1		1
Media exposure	Yes	0.79 (0.74, 0.84)*		0.74 (0.69, 0.79)*
	No	1		1
Wealth index	Poor	1		1
	Middle	0.98 (0.91, 1.05)		0.96 (0.89, 1.04)
	Rich	0.89 (0.82, 0.96)*		0.92 (0.84, 1.34)
Parity	Primiparous	1		1
	Multiparous	0.83 (0.69, 0.99)*		0.87 (0.73, 1.04)
	Grand multiparous	0.93 (0.74, 1.17)		1.05 (0.84, 1.33)
ANC visits attended during pregnancy	None	1		1
	1–3 visits	0.64 (0.59, 0.71)*		0.73 (0.63, 0.80)*
	4 or more visits	0.65 (0.59, 0.71)*		0.67 (0.62, 0.74)*
Place of delivery	Home	1		1
	Health facility	0.87 (0.81, 0.93)*		0.92 (0.85, 0.98)*
Table 6 (Continued)				
Currently breastfeeding	No	1		1
	Yes	1.09 (0.97, 1.20)		1.12 (1.01, 1.23)*
PNC checkup	No	1		1
	Yes	0.76 (0.71, 0.81)*		0.75 (0.70, 0.80)*
Sex of the child	Male	1		1
	Female	0.97 (0.92, 1.03)		0.97 (0.92, 1.03)
The current age of the child	6–11 months	1		1
	12–17 months	0.26 (0.25, 0.28)*		0.26 (0.24, 0.28)*
	18–23 months	0.14 (0.13, 0.16)*		0.14 (0.13, 0.16)*
Birth order	First-born	1		1
	Second to fourth	1.36 (1.13, 1.65)*		1.31 (1.09, 1.58)*
	Fifth or more	1.11 (0.87, 1.41)		1.02 (0.80, 1.29)
Preceding birth interval	< 24	1		1
	≥ 24	0.99 (0.92, 1.09)		0.97 (0.89, 1.06)
Number of under 5 children in the household	No child	1		1
	1–2	0.73 (0.57, 0.94)*		0.76 (0.59, 0.97)*
	3 and above	0.82 (0.64, 1.07)		0.77 (0.59, 1.00)
Place of residence	Rural		1	1
	Urban		0.82 (0.77, 0.86)*	0.99 (0.91, 1.07)
Community media exposure	Low		1	1
	High		0.97 (0.93, 1.03)	1.08 (0.99, 1.16)
Community poverty	Low		0.98 (0.92, 1.04)	0.95 (0.88, 1.03)
	High		1	1
Community illiteracy	Low		1.15 (1.09, 1.22)*	1.09 (1.02, 1.18)*
	High		1	1
Country category	Eastern		1.30 (0.99, 1.69)	0.76 (0.56, 1.05)
	Central		2.31 (1.77, 3.02)*	1.19 (0.86, 1.64)
	Western		2.52 (1.94, 3.28)*	1.62 (1.18, 2.22)*
	Southern		1	1

ANC = Antenatal Care; PNC = Postnatal Care

## Discussion

Appropriate complementary feeding practices address the fast-increasing nutrient gap between 6 and 24 months that contributes to undernutrition and its consequences. This study was conducted to determine the prevalence and associated factors of complementary feeding practices among mothers of children aged 6 to 23 months in SSA countries using the most recent DHS data. The findings of the current study revealed that the prevalence of appropriate complementary feeding practices among mothers of children aged 6 to 23 months in sub-Saharan African countries was 13.02% (95% CI: 12.75–13.29%). This finding was higher than studies conducted in western Ethiopia (9.91%) [31], Bensa district, south Ethiopia (8.6%) [32], northwest Ethiopia (10.6%) [33], another study in Ethiopia using 2019 mini EDHS (9.76%) [23], Malawi (7.7%) [22], Iseyin, Nigeria (10.0%) [18], and southwestern Nigeria (4.2%) [19]. The possible justification for this difference might be due to differences in study settings. All the previous studies were conducted in a single country or in a specific study area in a single country. Whereas the current study uses DHS data from 19 sub-Saharan African countries, which might increase the prevalence of appropriate complementary feeding practices. However, the current finding was lower than studies conducted in the south Kedida Gamela district, south Ethiopia (21%) [34], northern Ghana (29.8%) [20], rural northern Ghana (15.7%) [21], and India (51.3%) [17]. This difference might be due to sample size differences, differences in outcome variable definition, and differences in sociocultural and socioeconomic status. Unlike the previous studies, the current study uses revised indicators for assessing infant and young child feeding practices, and the outcome variable was defined using this indicator.

This study identified different factors significantly associated with appropriate complementary feeding practices, including maternal educational level, marital status of the mother, sex of the household head, total children ever born, media exposure, ANC visits attended during pregnancy, place of delivery, current breastfeeding, PNC checkup, the current age of the child, birth order, number of under five children in the household, community illiteracy, and country category. Maternal education was positively associated with appropriate complementary feeding practices. Similar findings were reported by studies conducted in northwest Ethiopia [33], western Ethiopia [31], and another study in Ethiopia using 2019 mini-EDHS [23], northern Ghana [20], and India [17]. This could be because educated mothers might read magazines, leaflets, and books that contain useful information about infant and young children’s feeding practices. They may also have a better chance of exposure to education about infant and young children’s feeding and child health services through mass media. Hence, mothers’ ability to make decisions and knowledge about their children’s nutritional desires may be improved. In addition, mothers who completed primary, secondary, and higher education have a high probability of securing paid jobs that increase their economic access to different food groups. Married women have higher odds of appropriate complementary feeding practices. This finding was in agreement with studies conducted in Kenya [35] and Gauteng Province [36]. This might be due to the absence of economic, social, and emotional support from a husband. Engaging fathers in activities like buying suggested foods, caring for and feeding children, and helping with household tasks improves complementary feeding practices [37].

The odds of appropriate complementary feeding practices were higher among female household heads compared with their counterparts. This finding was supported by a study conducted in the Amhara Region, northeast Ethiopia [38] and Kenya [39]. This might be attributed to the fact that mothers are mostly engaged in buying food in the household and are more accountable for the care of infants and young children than fathers. The study conducted in Kenya showed that mothers’ decisions on how to use family income were associated with more appropriate complementary feeding practices than fathers’ decisions on family income. This study also revealed that mothers who had less than nine children ever born were more likely to have appropriate complementary feeding practices. Similar results were reported by similar studies conducted in Southern Ethiopia [40], Northwest Ethiopia [41], and Taiwan [42]. This might be due to food insecurity in those households with more than nine children, and mothers may not get time to feed their children or prepare food. Households with large family sizes, food insecurity, and poor childcare practices were more likely to have malnourished children [43]. Similarly, mothers who had no media exposure were less likely to have appropriate complementary feeding practices. This finding was in agreement with studies conducted in the Amhara Region, northeast Ethiopia [38], Malawi [44], and Nepal [45]. Mothers who watch television, listen to the radio, and read newspapers or magazines may acquire essential information concerning complementary feeding practices. Mothers are more likely to adopt messages from mass media, as it is usually considered a reliable source of nutrition and health-related information. Mothers who did not attend ANC visits during pregnancy were less likely to have appropriate complementary feeding practices. The finding was supported by studies conducted in Ethiopia [23], Nigeria [19], and Bangladesh [46]. This could be because the information and counseling provided by health care professionals to mothers during their utilization of ANC services increases their knowledge about appropriate complementary feeding practices. This implies that the use of maternity services shall be promoted to improve infant and young children’s feeding practices.

Place of delivery was another significant factor in which mothers who delivered at home were less likely to have appropriate complementary feeding practices. A similar result was reported by a previous study conducted in Ethiopia [47]. This could be attributed to healthcare providers counseling about child health and nutrition during institutional delivery, which improves infant and young children’s feeding practices. The geographical region was also significantly associated with appropriate complementary feeding practices among mothers of children aged 6 to 23 in sub-Saharan African countries. The odds of appropriate complementary feeding practices were higher among mothers living in Western Africa. This might be related to the difference in the availability of health facilities, health extension workers, and maternal and child health services. In addition, the odds of appropriate complementary feeding practices were higher among mothers who currently breastfeed their children. This finding was supported by a study conducted in Ethiopia [23]. This might be due to complementary feeding being used to balance breast milk and ensure that a young child gets adequate calories, protein, and other nutrients to grow normally [48]. Complementary feeding is the initiation of other meals and liquids in addition to breast milk. Furthermore, increased odds of appropriate complementary feeding practices were found among mothers who had PNC checkups. This finding was consistent with similar studies conducted in Ethiopia [32–34] and Tanzania [49]. A possible explanation could be information delivery about appropriate complementary feeding practices during PNC follow-up, favoring appropriate infant and young child feeding. Information about appropriate complementary feeding practices and child care might be part of the counseling service that mothers received from health care professionals during their PNC visits. The current age of the child was another factor significantly associated with appropriate complementary feeding practices. Children aged 6–11 months were less likely to have appropriate complementary feeding practices. This finding was in agreement with studies conducted in Ethiopia [23, 31, 33], Nigeria [18], and Ghana [20]. This might be attributed to the misunderstanding of mothers that infants cannot digest foods like eggs, meat, and other fruits and vegetables compared with older children. In addition, older children are more likely to have an increased number of feeding times, which enhances the likelihood of appropriate complementary feeding practices. This implies that behavior change communication messages on the inclusion of diversified foods in the diet of children to mothers during their ANC and PNC visits should be emphasized.

In our study, birth order was significantly associated with appropriate complementary feeding practices. Children with a birth order of second to fourth had higher odds of appropriate complementary feeding practices. Previous studies conducted in Ethiopia [50], Aligarh [51], and Nepal [52] report similar findings. This might be related to increased awareness developed from previous feeding experiences among mothers with several births that might boost appropriate complementary feeding practices. In addition, low community illiteracy increases the odds of having appropriate complementary feeding practices by 1.09 times. This might be due to educating the community is educating mothers which indirectly has an impact on child health and nutritional status. Mothers who had no under five children in the household were less likely to have appropriate complementary feeding practices. This finding was supported by a study conducted in Uganda [53]. This might be due to a lack of previous exposure to appropriate complementary feeding practices.

### Strengths and limitations of the study

This study has the following strengths: First, the problem of the hierarchical nature of the DHS data is resolved as the study uses a multilevel mixed-effects model. Second, the most recent (2015–2020) DHS and region-wide survey data were used to help the concerned health authorities in sub-Saharan Africa set appropriate nutrition intervention strategies. Finally, the study uses the revised WHO indicators to determine the prevalence of appropriate complementary feeding practices in sub-Saharan Africa. The study has some limitations. There might be an introduction of recall and social desirability bias as meal frequency, dietary diversity, and the introduction of solid, semi-solid, and soft foods were measured based on mothers’ self-reports. The drawback of the secondary nature of the data was also expected.

## Conclusion

The prevalence of appropriate complementary feeding practices among mothers of children aged 6 to 23 months in Sub-Saharan Africa was relatively low. Individual-level factors like higher maternal educational level, female household head, having media exposure, attending more ANC visits during pregnancy, health facility delivery, currently breastfeeding, having PNC follow-up, and having a 12–23 months-old child have a positive association with appropriate complementary feeding practices. Whereas being unmarried, having a firstborn child, having more than nine children ever borne, and the absence of under-five children in the household have a negative association with appropriate complementary feeding practices. From community-level variables, low community illiteracy and living in the West Africa region had a positive association with appropriate complementary feeding practices among mothers of children aged 6 to 23 months in Sub-Saharan Africa. Health policymakers and other stakeholders working on child health are expected to design future interventions to improve complementary feeding practices targeting mothers who have no formal education, who have 6–11 months-old children, and who are unmarried. Women empowerment, increasing maternal health services accessibility, specifically antenatal and postnatal care services, promoting breastfeeding behavior, increasing media exposure in the household, and improving the proportion of health facility delivery are recommended to improve appropriate complementary feeding practice.

## Data Availability

The most recent data from the Demographic and Health Survey is publicly available online at (https://www.dhsprogram.com).

## List of abbreviations

ANC: Antenatal Care
AOR: Adjusted Odds Ratio
CF: Complementary Feeding
CI: Confidence Interval
DHS: Demographic and Health Survey
ICC: Intra-class Correlation Coefficient
MAD: Minimum Acceptable Diet
MDD: Minimum Dietary Diversity
MMF: Minimum Meal Frequency
MOR: Median Odds Ratio
PCV: Proportional Change in Variance
PNC: Postnatal Care
SSA: Sub-Saharan Africa
VIF: Variance Inflation Factor
WHO: World Health Organization
